# P-1538. Development of a Luminex Multiplexed Bead Based Assay to Detect IgG Vaccine Response to 32 Streptococcus pneumoniae Serotypes

**DOI:** 10.1093/ofid/ofaf695.1719

**Published:** 2026-01-11

**Authors:** Joe Douglas, Jerod Davidson, Michael Church, Jamie Nutt, Pam Morris, Steve Kleiboeker

**Affiliations:** Eurofins Viracor, Lenexa, KS; Eurofins Viracor, Lenexa, KS; Eurofins Viracor, Lenexa, KS; Eurofins Viracor, Lenexa, KS; Eurofins Viracor, Lenexa, KS; Eurofins Viracor, Lenexa, KS

## Abstract

**Background:**

*Streptococcus pneumoniae* is estimated to cause approximately 400,000 hospitalizations annually. Early mortality rate is as high as 26% for immunocompromised patients. In June 2024 a Pneumococcal 21-valent Conjugate Vaccine (P21-vCV) was approved by the FDA and includes serotypes not found in prior vaccines (Danish nomenclature): 15A, 15C, 16F, 23A, 23B, 24F, 31, and 35B. Detection of these new and existing serotypes is critical to determine immunity for immunocompromised patients.

**Figure 1.**
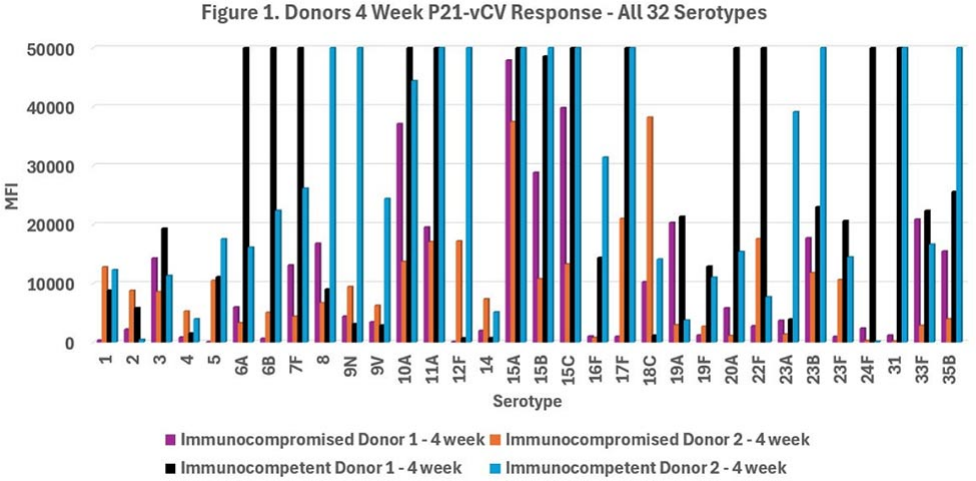
Donors 4 Week P21-vCV Response - All 32 Serotypes Post P21-vCV 4 week MFI IgG responses for all 32 serotypes for immunocompromised and immunocompetent donors.

**Figure 2.**
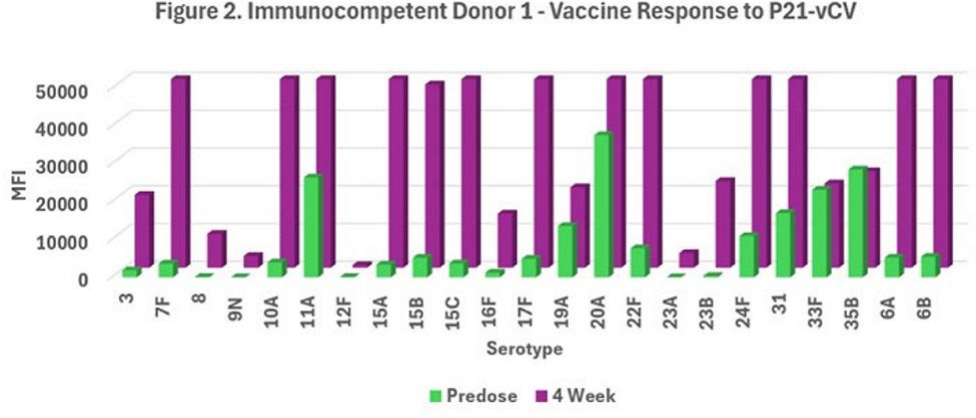
Immunocompetent Donor 1 - Vaccine Response to P21-vCV Immunocompetent Donor 1's MFI IgG response at Predose and 4 week post P21-vCV vaccination. 6B is not included in the P21-vCV vaccine, but the donor produced reactive IgG antibodies to both 6A and 6B.

**Methods:**

The international standard NIBSC 007sp and 23 unvaccinated reference range serum were tested. Serum from 2 immunocompetent and 2 immunocompromised individuals were collected at predose and 4 weeks after P21-vCV vaccination. IgG was detected using commercially available polysaccharides (1, 2, 3, 4, 5, 6A, 6B, 7F, 8, 9N, 9V, 10A, 11A, 12F, 14, 15A, 15B, 15C, 16F, 17F, 18C, 19A, 19F, 20A, 22F, 23A, 23B, 23F, 24F, 31, 33F, and 35B) activated with DMTMM and individually coupled to Luminex magnetic bead regions. A 32-plex bead pool was created. Samples were pre-processed for specificity, incubated with the bead pool, incubated with an anti-human IgG phycoerythrin antibody, and analyzed on a Intelliflex.

**Figure 3.**
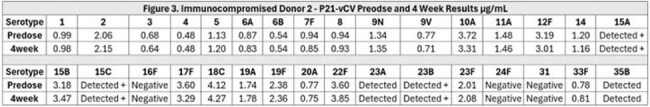
Immunocompromised Donor 2 - P21-vCV Predose and 4 Week Results µg/mL Immunocompromised donor 2 did not have a response to the vaccine from predose to 4 week. However, this patient was vaccinated to an earlier pneumococcal vaccine prior to immunosuppressive treatment and still has a >1.3 µg/mL IgG result to certain serotypes.

**Results:**

Immunocompetent donors had a median 12-fold increase in median fluorescent intensity (MFI) predose to 4 week.

Immunocompetent donor 1 had a MFI response to 23 of 32 serotypes.

Immunocompetent donor 2 had a MFI response to 22 of 32 serotypes.

Immunocompromised donor 1 had a lower MFI response compared to immunocompetent donors, but did respond to 13 of the 32 serotypes, Figure1.

Polysaccharides with similar conformation can produce cross-reactive IgG. In Figure 2, P21-vCV produced cross-reactive IgG to serotypes 6A and 6B.

Immunocompromised Donor 2 did not have a response to the vaccine. Prior vaccine immunity was detected at >1.3 µg/mL baselined from NIBSC 007sp, Figure 3. The reference range median MFI was 1,208 and a median value of 0.21 µg/mL. MFI results for new serotypes were “Negative” at < 1,208, “Detected” was 1,208-6,039, and “Detected +” was >6,040.

**Conclusion:**

Some serotypes can have cross-reactive IgG detection after vaccination. IgG was detected for all 32 serotypes. However, a commercially available international standard for the new serotypes is needed for accurate quantification.

**Disclosures:**

Joe Douglas, MS, Eurofins Viracor: Employee Jerod Davidson, B.A., Eurofins Viracor: Employee Michael Church, PhD, Eurofins Viracor: Employee Jamie Nutt, n/a, Eurofins Viracor: Employee Pam Morris, M.S., PMP, Pam Morris: Employee Steve Kleiboeker, PhD, Eurofins Viracor: Employee

